# Effect of aqueous and hydro-alcoholic extracts of lettuce (Lactuca sativa) seed on testosterone level and spermatogenesis in NMRI mice 

**Published:** 2014-01

**Authors:** Akram Ahangarpour, Ali Akbar Oroojan, Maryam Radan

**Affiliations:** 1 *Health Research Institute, Diabetes Research Center, Department of Physiology, Ahvaz Jundishapour University of Medical Sciences, Ahvaz, Iran.*; 2 *Student Research Committee, Ahvaz Jundishapur University of Medical Sciences, Ahvaz, Iran.*; 3 *Department of Physiology, Ahvaz Jundishapur University of Medical Sciences, Ahvaz, Iran.*

**Keywords:** *Lactuca sativa seed*, *Sperm*, *Testosterone*, *Testis*

## Abstract

**Background:** One of the considerable uses of lettuce (Lactuca sativa) seed in traditional medicine has been to reduce semen, sperm and sexuality.

**Objective: **The aim of this study was to investigate the effects of aqueous and hydro-alcoholic extracts of lettuce seed on testosterone level and spermatogenesis.

**Materials and Methods:** In this experimental study 24 adult male NMRI mice weighing 20-25gr were purchased. Animals were randomly divided into 4 groups: controls, hydro-alcoholic (200 mg/kg) and aqueous extracts (50, 100mg/kg). The extracts were injected intraperitoneally once a day for 10 consecutive days. 2 weeks after the last injection, the mice were anaesthetized by ether and after laparatomy blood was collected from the heart to determine testosterone by ELISA assay kit. Then testis and cauda epididymis of all animals were removed for analyzing testis morphology and sperm count and viability.

**Results:** Testis weight in hydro-alcoholic and aqueous extracts 100 mg/kg (p=0.001) and aqueous extract 50 mg/kg (p=0.008) groups was increased. Sperm viability in hydro-alcoholic (p=0.001) and aqueous extracts 50 (p=0.026), 100 mg/kg (p=0.045) groups was decreased, Also the results showed a significant decrease in sperm count in hydro-alcoholic (p=0.035) and aqueous extracts 50 mg/kg (p=0.006) groups in comparison with control group. Also there was a significant increase in serum level of testosterone in aqueous extract 50 mg/kg group in comparison with control (p=0.002) hydro-alcoholic (p=0.001) and aqueous extracts 100 mg/kg (p=0.003) groups.

**Conclusion:** Present results demonstrated that hydro-alcoholic and aqueous 50 mg/kg extracts of lettuce seed have antispermatogenic effects, also aqueous extract 50 mg/kg increased serum level of testosterone in mice. Therefore we can suggest that lettuce seed could be a potential contraceptive agent.

This article extracted from M.Sc. student research project. (Ali Akbar Oroojan)

## Introduction

In the developing world, the population is increasing rapidly. About 3/4 of the 3.2 billion will increase in the world’s population by 2025 that it’s expected to take place in developing countries. It is predicted that global population will arrive to 7.8-12 billion by 2050 (1). Overpopulation can be a serious problem causing much economic, social and environmental resources problems. The world’s population has increased exponentially since the industrial revolution is the main cause of modern technologies and medicines available (1). The assurance of long term use of contraceptives is controversial. To this effect, World Health Organization is trying to find a safe, affordable and socially acceptable alternative (2). In the last decade, there has been a great progress in the pharmacological evaluation of medicinal plants especially in the third world that could be a useful contraceptive and fertility control agents and possible replacement for hormonal contraceptives. Nowadays focus on herbal research has increased in the world and many plants are known with contraceptive properties (3). 

According to Maurya *et al* review, 48 out of 72 traditionally employed antifertility medicinal plants showed antifertility effects (4). But a few of plants have this effect in the male reproductive system. *Azadirachta indica* leaves, *Aristolochia indica* root, *Ananas comosus* unripe fruit, *Embelia ribes* fruits, *Carica papaya* seeds, flowers of *Hibiscus rosa sinesis*, *Malvasicus conzatti* and *Aristolochia indica* root have showed antispermatogenetic effect in mice. Spermicidal activity in many plants has been investigated. Spermicidal saponins from *Sapindus mukrossi* fruit, *Schefflera capitata*, *Pittosporum nilgherense* and *Polemonium coeruleum* have been characterized (5). 

Lettuce (*Lactuca sativa*) belongs to Asteraceae (Compositae) family, tribe Cichorieae and exhibits excellent medicinal properties (6). This plant is a rich source of carotene, vitamin C and E. Lettuce herb possesses sedative, analgesic, anticonvulsant, hypoglycemic and antifungal properties. Presence of high amount of carotene, Vitamin C and Vitamin E reflects noticeable antioxidant properties (7). Traditional uses of lettuce seed in Iran were applied to relieve inflammation, gastrodynia and osteodynia. It was demonstrated that methanolic extract of the seed, contains triterpenoids, saponins and simple phenols that possesses antinociceptive and anti-inflammatory effects (8). 

Xu *et al* demonstrated that lettuce seeds have a new flavonol glycoside with a rare structure type, lactuca sativo side A, together with 3 known compounds, japonica A, isoquercitrin and caffeic acid (9). Spermatogenesis is the process of differentiation in germ cells those ultimates to production of spermatozoa. Initiation of spermatogenesis occurs at the time of puberty and its associated with the transition from a relatively hypogonadotropic state in the pubertal phase of development to the eugonadotropic state in adulthood. Spermatogenesis compartmentalized within the blood-testis barrier. It is essentially under FSH regulation, and involves close cell-to-cell interaction between Sertoli and germ cell at all stage of spermatogenic maturation (10). The in vivo study on Swiss male albino mice showed that ethanol extract of stem bark of Dalbergia sissoo Roxb have a significant decrease in sperm motility and sperm count in the epididymis (11). 

Furthermore aqueous extract of Aframomum longiscapum seeds exhibited a negative effect on male rat fertility by reducing sperm count, motility and viability (12). Gupta *et al* showed that isolated saponins from Albizia lebbeck bark have a significant decrease in spermatid, spermatocytes, spermatogonia, sperm motility and sperm density, also Rajasekaran *et al* illustrated that antifungal saponin of ethyl acetate fraction of Mollugo pentaphylla has a potential spermicidal effect and mechanism of its action involves sperm membrane damage by increased lipid peroxidation (13, 14). 

Since, lettuce seed’s extract contains saponin, and it was demonstrated that this compound has negative effects on reproductive system of male rats and one of the considerable uses of brewed lettuce seed in traditional medicine, is to reduce semen, sperm and sexuality, we attempted to investigate the effects of aqueous and hydro-alcoholic extracts of lettuce seed on testosterone and spermatogenesis (8, 13, 15). Since methanolic extract of the seeds contains triterpenoids, phenolic carboxylic acids and saponins, we utilized this extract for looking negative effects of saponin on spermatogenesis (8). 

## Materials and methods

**Plant extraction**

In this experimental study, dry lettuce (*Lactuca sativa*) seed were purchased from Ahvaz green-grocery and after authentication by botany proficient were powdered by grinder. 

**Aqueous extract**

For the sake of making lettuce seed aqueous extract, according to traditional utilization 50g of seed’s powder was added to 200ml boiling distilled water for 15 min (16). Then mixture was filtered with Whatman No 1 filter paper and centrifuged with 3500 rpm for 20 min. After that, solution dried at room temperature to obtain powder and this extract’s powder was stored at refrigerator until being used (16). 

**Hydro-alcoholic extract**

The fine 50g powder obtained was firstly macerated in 200 ml (70-30 methanol and distilled water) for 72 hr. The mixture was filtered with Whatman grade No. 1 filter paper and centrifuged with 3500 rpm for 20 min. At the end, solvent was removed and the solid remainder was collected and dried at room temperature and the extracted powder was kept at 4^o^C until used (17). 

**Animal’s preparation**

24 adult male NMRI mice weighing 20-25gr were purchased from Ahvaz Jundishapur University of Medical Sciences )AJUMS) animal facility. Animals used in this study were treated in accordance with principals and guidelines on animals care of AJUMS and were kept at 20-24ºC under 12 h light/dark cycle and were allowed free access to tap water and commercial chow. Animals were divided into 4 groups, including 6 mice in each group: (controls (received saline 0.9%), mice injected with hydro-alcoholic (200 mg/kg) and aqueous extracts (50, 100 mg/kg) of lettuce seed) (18, 19). 

**Toxicity assessment**

In Sayyah *et al* study, dose of 6g/kg methanolic extract illustrated toxic effect in mice and the incidence of mortality was noted up to 24 h after administration and the effective dose of this extract was between 50 and 500 mg/kg, therefore we decided to use one dose (200 mg/kg) of methanolic extract in this research but there was no study in aqueous extract of lettuce seed, so we administrated 3 doses (50, 100, 200 mg/kg) of this extract (8). After first injection of aqueous extract (200 mg/kg), all of the animals were died after 24h. 

Therefore this dose of extract was eliminated of the research. These differences between tolerance of hydro alcoholic 200 mg/kg and intolerance of aqueous extracts 200 mg/kg are probably referring to the side effects and toxic effects that is presence in aqueous extract of the seeds. Experimental protocols: Spermatogenesis in the mouse can be divided into 16 stages and requires approximately 229h (9.5 days) to complete. Therefore the extracts were administered intraperitoneally once a day for 10 consecutive days in test groups (20). 

2 weeks after the last injection, the mice were anaesthetized by ether and after laparotomy blood was collected from the heart (21). Thereafter testes in control and experiments groups were immediately removed. The weight, length and width of testis in each group were analyzed also testicular volume was calculated according to the following formula: 

$\mathrm{volume}=\left(\frac{D^{2}}{4}\times \pi \right)L\times K$

(L: length, D: width, K: 0.9, π: 3.14) (22). 

After macroscopic observations, for the sake of sperm computation, cauda epididymis of all animals was dissected and transferred into 1.5 ml normal saline 0.9% and cut to small slices. Then spermatozoa were dispersed into the normal saline solution. After that a drop of solution was transferred into each chamber of Neubauer hemocytometer (*HBG*. *Company*, *Germany*) (Tiefe depth profondeur 0.100 mm and 0.0025 mm^2^ area) and sperm count was manually counted in white blood cell grids under a light microscope (Olympus light microscope Tokyo, Japan) and data were expressed as the count of sperm per ml. 

The eosin 1% staining (Merk Chemical Co, Germany) were used for evaluation of live (unstained) and dead (red stained) sperm. After addition of the stain on each chamber of Neubauer hemocytometer and leave for 30 seconds, a total of spermatozoa were counted within 2 min (23). 

**Serum testosterone measurements**

After blood collection from the heart, specimens were centrifuged. Then serum concentration of testosterone was measured by using a testosterone ELISA assay kit (DRG Instruments GmbH, Germany). The sensitivity of hormone detection per assay tube was 0.083ng/ml.

**Statistical analysis**

The results were statistically analyzed by SPSS software with one-way ANOVA and post hoc LSD tests. The results were expressed as mean±SEM (standard error of means) and p<0.05 was considered significant. 

## Results

**Effect of** **aqueous and hydro-alcoholic extracts of lettuce seed on weight (g) and testis parameters**

The obtained results in this study showed that there was no significant difference in testis weight before and after of experimental period also there was no significant difference in testis length, width and volume between groups, but testis weight (mg) increased in hydro-alcoholic and aqueous extracts 100 mg/kg (p=0.001) and aqueous extract 50 mg/kg (p=0.008) in comparison with control group (Table I).

**Effect of aqueous and hydro-alcoholic extracts of lettuce seed on sperm viability and count**

**Figure 1:**
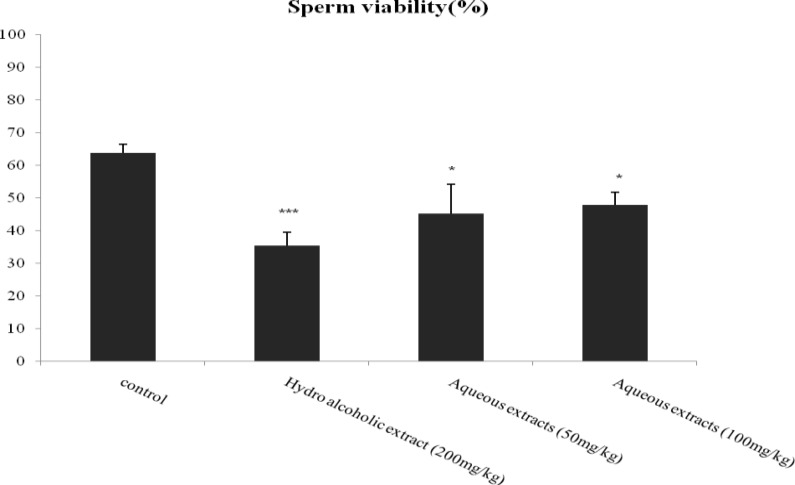
Effect of aqueous and hydro-alcoholic extracts of lettuce seed on sperm viability.

**Figure 2:**
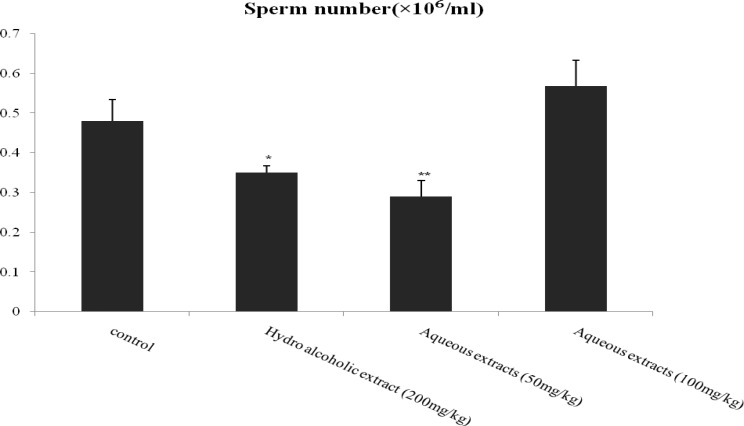
Effect of aqueous and hydro-alcoholic extracts of lettuce seed on sperm count.

The percentage of viable sperm in hydro-alcoholic (p=0.001) and aqueous extracts 50 (p=0.026), 100 mg/kg (p=0.045) of lettuce seed decreased significantly in comparison with control group (Figure 1). Results of this study showed a significant decrease in epididymal sperm count (×10^6^/ml) in hydro-alcoholic (p=0.035) and aqueous extracts 50 mg/kg (p=0.006) of lettuce seed in comparison with control group (Figure 2).

**Effect of aqueous and hydro-alcoholic extracts of lettuce seed on testosterone**

**Figure 3:**
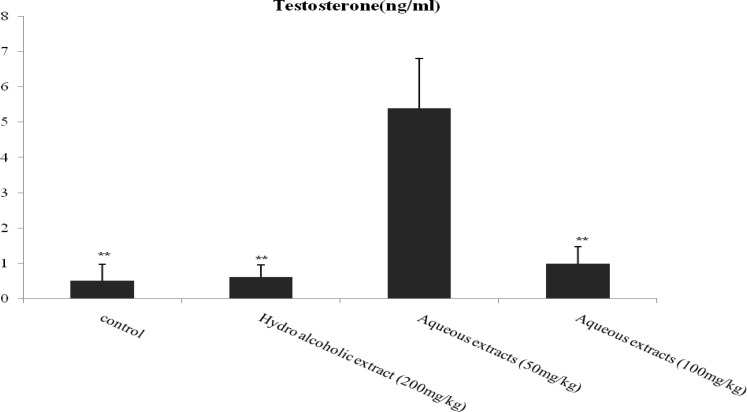
Effect of aqueous and hydro-alcoholic extracts of lettuce seed on Testosterone.

The results of this study illustrated that serum level of testosterone (ng/ml) in the treatment group with aqueous extract 50 mg/kg increased significantly in comparison with control (p=0.002), hydro-alcoholic (p=0.001) and aqueous extract 100 mg/kg (p=0.003) groups (Figure 3).

**Table I:** Effect of aqueous and hydro-alcoholic extracts of lettuce seed on weight and testis morphology

**Group**	**Animal weight** **Before** **(g)**	**Animal weight** **After** **(g)**	**Testis weight** **(mg)**	**Testis length** **(mm)**	**Testis width** **(mm)**	**Testis volume** **(cm3)**
Control	24.26 ± 1.89	28.41 ± 2.05	63.33 ± 3.07	7 ± 0.25	4.33 ± 0.21	0.095 ± 0.012
Hydro-alcoholic extract (200 mg/kg)	24.72 ± 0.91	29.53 ± 1.16	78.33 ± 3.11*	7.33 ± 0.16	4.22 ± 0.14	0.093 ± 0.007
Aqueous extract (50 mg/kg)	24.1 ± 0.77	28.5 ± 1.39	75 ± 1.29**	6.5 ± 0.34	4.33 ± 0.21	0.087 ± 0.009
Aqueous extract (100 mg/kg)	25.41 ± 2.24	31.06 ± 2.36	79.16 ± 1.53***	7.33 ± 0.33	4.5 ± 0.22	0.106 ± 0.013

n=6,

** =p<0.01,

*** =p<0.001

* = difference between control group with other groups.

(n=6, *=p<0.05, ***=p<0.001). *= difference between control group with other groups.

(n=6, *=p<0.05, **=p<0.01). *= difference between control group with other groups.

(n=6, **=p<0.01). *= difference between aqueous extract (50mg/kg) group with other groups.

## Discussion

The results of this research showed that aqueous and hydro-alcoholic extracts of lettuce seed increased testis weight which was accompanied by an increase in the serum levels of testosterone in aqueous extract 50 mg/kg group. In Raji *et al* study, they have been reported that *Morinda lucida* leaf extract could impair reproductive activities in male albino rats also may cause an increase in the weight of the testes and serum levels of testosterone. Similar changes were demonstrated with the extract of *Zingiber officinale* and *Pentadiplendra brazzeana* in rats that these results are agreement with our findings in aqueous extract (50 mg/kg) in this study (24). 

All of the utilized extracts in this research elucidate that lettuce seed has spermicidal effect, because sperm viability was decreased in all extract group’s animals in comparison with control group. The decline in viability of spermatozoa might be due to the spermicidal action of the extracts. Also the present study clearly demonstrates that epididymal sperm count following hydro-alcoholic and aqueous extracts 50 mg/kg of lettuce seed will be decreased significantly. One of the important gonadal steroid hormones is testosterone that adjusts spermatogenesis (25). Spermatogenesis is dependent on gonadotropin stimulation and testosterone in turn stimulates spermatogenesis through the androgen receptors (10). 

Flutamide and related drugs are nonsteroidal competitive antagonists of androgen receptors. These drugs are used to decrease the action of endogenous androgen by inhibition of binding at its receptor. Also flutamide can ultimate surge of testosterone synthesis caused by the initial agonistic action of the GnRH agonist (26). In Yu *et al* study, it was demonstrated that flutamide treatment markedly increased serum level of testosterone and this effect was probably due to suppression on feedback inhibition of androgens on the anterior pituitary gland, then secondarily of LH secretion in pituitary gland and a blockade of androgen receptors, resulting in stimulating the secretion of testosterone. Also this drug decreased sperm count and sperm viability in the cauda epididymis (27). 

According to the results of aqueous extract 50 mg/kg on serum level of testosterone, sperm and sperm viability in this study, we can suggest that these results are corresponded with flutamide effects and mechanism of action on testosterone, sperm and sperm viability. In Li *et al* research it was indicated that seeds of *Camellia oleifera* decreased percent of live sperm and sperm count moreover it expressed that, *Camellia oleifera* saponins was the main reason of these effects (28). Therefore according to outcome of this study and presence of saponins in lettuce seed, we can suggest that, this compound probably induce antispermatogenic and spermicidal effects in treated animals. The results showed sperm count in mice that received aqueous extracts 100 mg/kg unlike mice receiving 50 mg/kg didn’t significantly change. 

Therefore we can suggest that the effect of this extract is in a dose dependent manner. In Ahmadi *et al* research it was illustrated that the effect of *Salvia officinalis* extract 150 or 200 mg/kg could not significantly change serum creatine kinase level but 100 mg/kg dose of this extract decreased serum level of creatine kinase significantly, thus the effects of this extract was a dose dependent manner (29). 

Also in Pourmehdi Rad *et al* study *Matricaria recutita* extract 10, 30 mg/kg induced anxiolytic effect but 50 mg/kg of this extract did not show anxiolytic effects in comparison with control group, so the effects of this extract showed a dose dependent manner, therefore these effects are agreement with our results in the present study (30). 

## Conclusion

In conclusion, we have demonstrated that hydro-alcoholic and aqueous extracts 50 mg/kg of lettuce seed have antispermatogenic and spermicidal effects by decreasing in sperm count and sperm viability also aqueous extract 50 mg/kg increased serum level of testosterone in mice and these effects were in dose dependent manner. 

Therefore we can suggest that lettuce seed could be a potential contraceptive agent, and the contraceptive potential of this seed is also inferred from its adverse effect on viability and count of the sperm. Moreover, since aqueous extract 50 mg/kg showed the same effect of flutamide on serum level of testosterone and sperm count we can suggest to do further studies are required to clarify the similarity between this extract and flutamide on male reproductive system.
